# Digital Dentistry in Clinical Practice: A Scoping Review of Current Capabilities and Future Directions

**DOI:** 10.1016/j.identj.2025.109296

**Published:** 2025-11-29

**Authors:** Walter Yu Hang Lam, Zhaoting Ling, Kaijing Mao, Ji-Man Park, Amirali Zandinejad, Adriana da Fonte Porto Carreiro, Francesco Guido Mangano, Jeffrey A. Platt, Falk Schwendicke

**Affiliations:** a Restorative Dental Sciences, Faculty of Dentistry, the University of Hong Kong, 34 Hospital Rd, Sai Ying Pun, Hong Kong Special Administrative Region, China; b Department of Prosthodontics and Dental Research Institute, Seoul National University, Seoul, Republic of Korea; c Implant Dentistry Associates of Arlington, Texas, USA; d Department of Prosthodontics, School of Medicine and Dentistry, University of Rochester, New York, USA; e Department of Dentistry, Federal University of Rio Grande Do Norte, Natal, Brazil; f Preventive Dentistry and Orthodontics, I. M. Sechenov First State Medical University, Moscow, Russian Federation; g Department of Biomedical and Applied Sciences, Division of Dental Biomaterials, Indiana University School of Dentistry, Indiana, USA; h Clinic for Conservative Dentistry, Periodontology and Digital Dentistry, University Hospital of the Ludwig-Maximilians-University Munich, Munich, Germany

**Keywords:** Artificial intelligence, Computer-aided design, Computer-aided manufacturing, Digital technology, Digital health, Three-dimensional imaging

## Abstract

Digital technologies are transforming oral healthcare by enhancing prevention, diagnostics, treatment, and maintenance procedures. However, few comprehensive reviews have synthesized their clinical applications across dental disciplines. This scoping review maps the clinical applications of digital dentistry and informed the development of a 2025 FDI Policy Statement that will guide stakeholders in recognizing both significant advances and ongoing challenges. A systematic search of PubMed, Embase, and Web of Science identified 407 eligible articles. Applications clustered into 2 domains: Disease prevention and diagnosis – preventive dentistry (n = 39), cariology (n = 26), and periodontology (n = 16), and Management of disease consequences and patient care – prosthodontics (n = 127), oral and maxillofacial surgery (n = 112), orthodontics (n = 26), and perioperative management (n = 61). Digital dentistry encompasses artificial intelligence, computer-aided design-computer-aided manufacturing (CAD-CAM) technologies, computer-assisted surgery systems, digital imaging, teledentistry, and related devices and systems. Evidence supporting digital applications should be critically evaluated, and professional judgment must remain central to patient care. Advancing the field will require more standardized, high‑quality data and clinical research to establish robust evidence of real‑world impact.

## Introduction

The introduction of computer-aided design-computer-aided manufacturing (CAD-CAM) technology into prosthodontics in the 1970s marked a pivotal advance in dental practice and paved the way for contemporary digital dentistry.^1^ Early applications enabled the fabrication of crowns using workflows that began with optical impressions and concluded with automated milling.^2^ Over subsequent decades, ongoing technological innovation and the integration of digital tools have transformed digital dentistry into an essential component of modern care. Reflecting this progress, the International Association for Dental, Oral, and Craniofacial Research (IADR) established the Digital Dentistry Research Network in 2022 to advance research in this rapidly evolving field. Likewise, the International Organization for Standardization (ISO), through Technical Committee (TC) 106 (Dentistry), Subcommittee 9, has promoted the standardization of dental CAD-CAM systems. Moreover, an international group of clinicians and researchers founded the Digital Dentistry Society, which aims to promote and advance the science and practice of digital dentistry.

Digital dentistry broadly refers to the application of digital technologies to address oral health challenges, as outlined in World Health Organization (WHO) publications.^3^^,^^4^ Today, it encompasses a wide array of innovations, including digital scanners and imaging tools, CAD-CAM systems, computer-assisted surgery, mobile health applications, and, more recently, artificial intelligence (AI). These technologies have transformed dental practice by improving accuracy, expediting treatment, enhancing communication, enabling greater customization, supporting more predictable planning, and optimizing patient care.^5^, ^6^, ^7^, ^8^

Despite these advances, integrating digital dentistry into routine care remains challenging and should be evidence-based, outcome-driven, high quality, patient-centred, ethical, fair, and inclusive. Robust governance and clear legal and regulatory frameworks must safeguard privacy and data security while enabling safe collection, storage, and responsible access to data for care, research, and innovation; informed consent for primary and secondary data use should be explicit and documented. Data quality is critical – biased or incomplete datasets can undermine performance, especially in AI – so evidence should be critically appraised and supported by standards that ensure quality, safety, effectiveness, interoperability, sustainability, and alignment with primary healthcare and global oral health strategies. Interoperable, user-friendly tools accessible to both providers and patients are essential to promote equity. To avoid overreliance on technology, continuous professional development and comprehensive curricula across undergraduate, postgraduate, and continuing education should strengthen clinical judgment and responsible patient management. Finally, the rapid pace of innovation can raise costs and limit access, underscoring the need for sustainable, outcome-focused, and equitable adoption.

To address these concerns, the World Dental Federation (FDI) issued a Policy Statement in 2025 to provide guidance for stakeholders on the responsible adoption of digital technologies while minimizing associated risks (https://fdiworlddental.org/digital-dentistry). This scoping review synthesizes the clinical applications of digital dentistry, highlights their value in practice, identifies priorities for future development, and provides an in-depth analysis of current and emerging digital technologies in dentistry.

## Materials and methods

A comprehensive search of electronic databases, including PubMed, Embase, and Web of Science, was conducted from inception to October 2024 and updated in June 2025, following PRISMA-ScR guidelines.^9^ This review protocol was registered with the Open Science Framework (reference number: osf.io/6crn9). The search strategy combined keywords related to dentistry, digital technologies, artificial intelligence, and clinical studies.

The search string used was: (dentistry OR dental OR tooth OR teeth OR oral health) AND ((digital) OR (artificial intelligence) OR (machine learning) OR (deep learning) OR (intraoral scanner) OR (facial scanner) OR (CAD) OR (CAM) OR (3D) OR (virtual patient) OR (virtual articulator) OR (digital smile design) OR (mobile) OR (smartphone) OR (teledentistry) OR (computer-aided) OR (computer-assisted) OR (virtual reality)) AND (elderly OR adult OR adolescent OR child OR patient OR participant OR subject). Filters were applied to limit results to clinical study types, including clinical trials, comparative studies, multicentre studies and observational studies.

The inclusion criteria were as follows:

1. English-language original articles reporting clinical applications of digital dentistry in humans. Nonclinical articles – such as animal or materials research, surveys and reviews – were excluded.
2. Studies unrelated to clinical practice or outcome improvement, including those where digital technologies were used solely for dental education or as measurement tools to evaluate clinical outcomes, were excluded.
3. Although digital radiology (including 2D intraoral and panoramic radiographs and 3D cone-beam computed tomography [CBCT]) can be considered part of digital dentistry, this area was excluded as it is already routine in many regions.

Two reviewers (Z.L. and K.M.) independently screened titles and abstracts, and assessed full-text articles for eligibility using the Covidence systematic review software.^10^ Selection of studies was based on consensus; disagreements were resolved with a third reviewer (W.L.). For each included study, the following data were extracted: general study information (first author, year of publication, country/region, and discipline), study population, interventions (digital technologies used and key applications), and reported outcomes. Included studies were analysed and categorized by dental discipline. Within each discipline, studies were arranged first by the sequence from prevention to diagnosis and risk prediction, then by clinical workflow (eg, planning, treatment, prosthesis fabrication), and finally alphabetically.

## Results

**Fig. 1:**
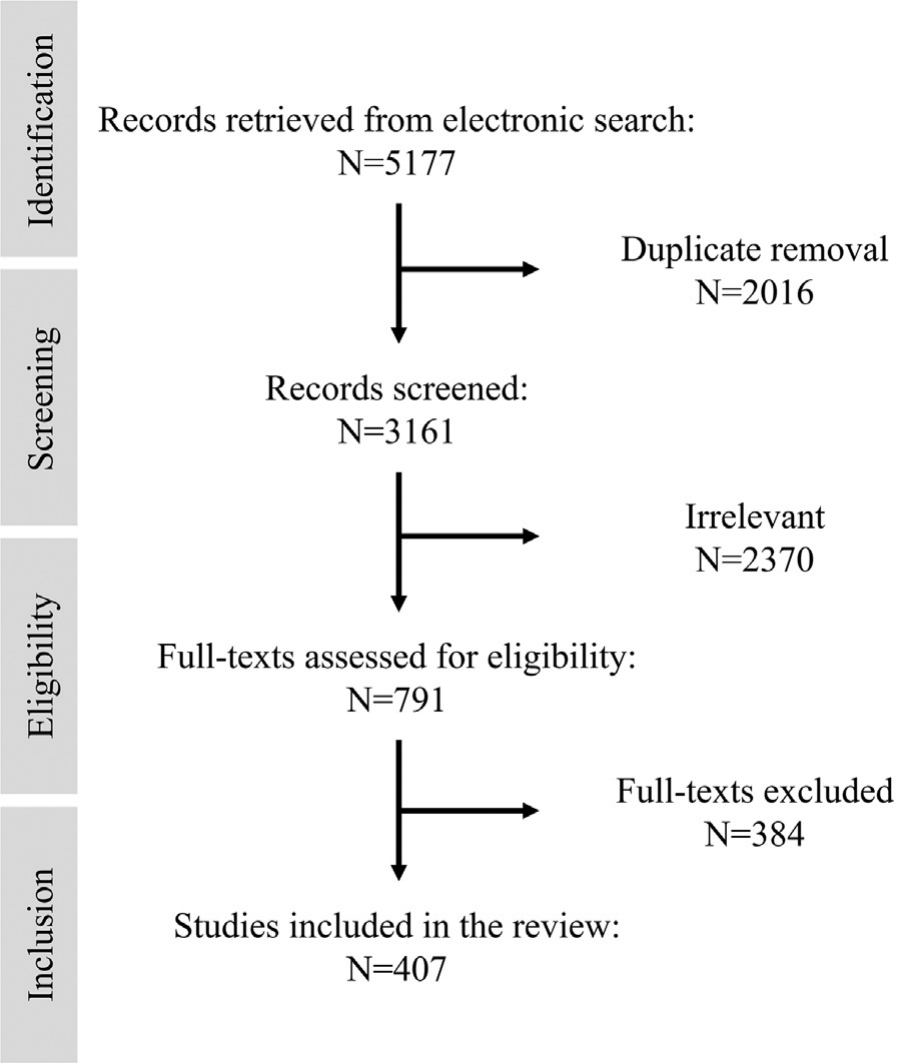
Flowchart illustrating the screening process and inclusion of studies in this scoping review.

**Fig. 2:**
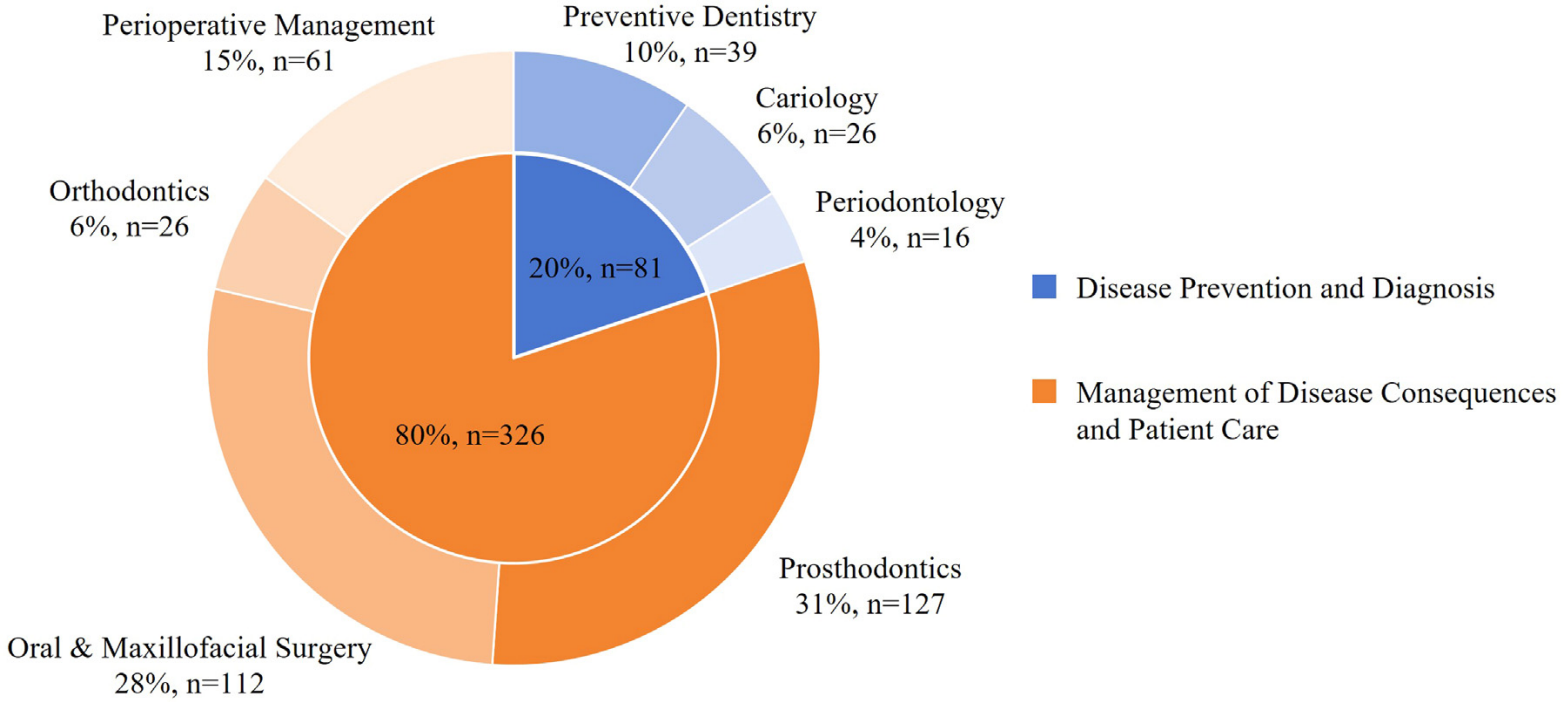
Distribution of studies across various.

The initial database search yielded 5177 articles, which were reduced to 3161 after the removal of duplicates. Title and abstract screening excluded 2370 articles as irrelevant. Of the remaining articles, 384 were excluded after full-text assessment for the following reasons: abstract only/full-text not available (8 articles), non-English language articles (15 articles), not clinical trials (265 articles), or not related to clinical practice or outcome improvement (96 articles). Ultimately, 407 articles were included in the review (Figure 1). The included studies covered a range of dental disciplines and were clustered into 2 domains (Figure 2): (1) disease prevention and diagnosis – preventive dentistry (n = 39),^11^, ^12^, ^13^, ^14^, ^15^, ^16^, ^17^, ^18^, ^19^, ^20^, ^21^, ^22^, ^23^, ^24^, ^25^, ^26^, ^27^, ^28^, ^29^, ^30^, ^31^, ^32^, ^33^, ^34^, ^35^, ^36^, ^37^, ^38^, ^39^, ^40^, ^41^, ^42^, ^43^, ^44^, ^45^, ^46^, ^47^, ^48^, ^49^ cariology (n = 26),^50^, ^51^, ^52^, ^53^, ^54^, ^55^, ^56^, ^57^, ^58^, ^59^, ^60^, ^61^, ^62^, ^63^, ^64^, ^65^, ^66^, ^67^, ^68^, ^69^, ^70^, ^71^, ^72^, ^73^, ^74^, ^75^ and periodontology (n = 16)^76^, ^77^, ^78^, ^79^, ^80^, ^81^, ^82^, ^83^, ^84^, ^85^, ^86^, ^87^, ^88^, ^89^, ^90^, ^91^; and (2) management of disease consequences and patient care – prosthodontics (n = 127),^92^, ^93^, ^94^, ^95^, ^96^, ^97^, ^98^, ^99^, ^100^, ^101^, ^102^, ^103^, ^104^, ^105^, ^106^, ^107^, ^108^, ^109^, ^110^, ^111^, ^112^, ^113^, ^114^, ^115^, ^116^, ^117^, ^118^, ^119^, ^120^, ^121^, ^122^, ^123^, ^124^, ^125^, ^126^, ^127^, ^128^, ^129^, ^130^, ^131^, ^132^, ^133^, ^134^, ^135^, ^136^, ^137^, ^138^, ^139^, ^140^, ^141^, ^142^, ^143^, ^144^, ^145^, ^146^, ^147^, ^148^, ^149^, ^150^, ^151^, ^152^, ^153^, ^154^, ^155^, ^156^, ^157^, ^158^, ^159^, ^160^, ^161^, ^162^, ^163^, ^164^, ^165^, ^166^, ^167^, ^168^, ^169^, ^170^, ^171^, ^172^, ^173^, ^174^, ^175^, ^176^, ^177^, ^178^, ^179^, ^180^, ^181^, ^182^, ^183^, ^184^, ^185^, ^186^, ^187^, ^188^, ^189^, ^190^, ^191^, ^192^, ^193^, ^194^, ^195^, ^196^, ^197^, ^198^, ^199^, ^200^, ^201^, ^202^, ^203^, ^204^, ^205^, ^206^, ^207^, ^208^, ^209^, ^210^, ^211^, ^212^, ^213^, ^214^, ^215^, ^216^, ^217^, ^218^ oral and maxillofacial surgery (n = 112),^219^, ^220^, ^221^, ^222^, ^223^, ^224^, ^225^, ^226^, ^227^, ^228^, ^229^, ^230^, ^231^, ^232^, ^233^, ^234^, ^235^, ^236^, ^237^, ^238^, ^239^, ^240^, ^241^, ^242^, ^243^, ^244^, ^245^, ^246^, ^247^, ^248^, ^249^, ^250^, ^251^, ^252^, ^253^, ^254^, ^255^, ^256^, ^257^, ^258^, ^259^, ^260^, ^261^, ^262^, ^263^, ^264^, ^265^, ^266^, ^267^, ^268^, ^269^, ^270^, ^271^, ^272^, ^273^, ^274^, ^275^, ^276^, ^277^, ^278^, ^279^, ^280^, ^281^, ^282^, ^283^, ^284^, ^285^, ^286^, ^287^, ^288^, ^289^, ^290^, ^291^, ^292^, ^293^, ^294^, ^295^, ^296^, ^297^, ^298^, ^299^, ^300^, ^301^, ^302^, ^303^, ^304^, ^305^, ^306^, ^307^, ^308^, ^309^, ^310^, ^311^, ^312^, ^313^, ^314^, ^315^, ^316^, ^317^, ^318^, ^319^, ^320^, ^321^, ^322^, ^323^, ^324^, ^325^, ^326^, ^327^, ^328^, ^329^, ^330^ orthodontics (n = 26),^331^, ^332^, ^333^, ^334^, ^335^, ^336^, ^337^, ^338^, ^339^, ^340^, ^341^, ^342^, ^343^, ^344^, ^345^, ^346^, ^347^, ^348^, ^349^, ^350^, ^351^, ^352^, ^353^, ^354^, ^355^, ^356^ and perioperative management (n = 61).^357^, ^358^, ^359^, ^360^, ^361^, ^362^, ^363^, ^364^, ^365^, ^366^, ^367^, ^368^, ^369^, ^370^, ^371^, ^372^, ^373^, ^374^, ^375^, ^376^, ^377^, ^378^, ^379^, ^380^, ^381^, ^382^, ^383^, ^384^, ^385^, ^386^, ^387^, ^388^, ^389^, ^390^, ^391^, ^392^, ^393^, ^394^, ^395^, ^396^, ^397^, ^398^, ^399^, ^400^, ^401^, ^402^, ^403^, ^404^, ^405^, ^406^, ^407^, ^408^, ^409^, ^410^, ^411^, ^412^, ^413^, ^414^, ^415^, ^416^, ^417^ Among the included studies, the leading contributing countries were Italy (10%), the United States (9%), and China (9%), followed by India (8%) and Germany (7%).

## Discussion

### Clinical applications of digital dentistry across dental disciplines

#### Disease prevention and diagnosis

Disease prevention and early diagnosis of oral diseases were prominent, with risk prediction enabling personalized prevention and care. Approximately 20% of the included studies focused on this domain, with notable emphasis on oral health promotion (preventive dentistry) and a particular concentration on 2 major dental diseases: dental decay (cariology) and periodontal diseases (periodontology).

##### Preventive dentistry

Preventive dentistry – traditionally categorized as primary, secondary, and tertiary prevention – has increasingly incorporated digital technologies.^418^ Two key technologies – teledentistry and artificial intelligence – have been used across prevention levels. Teledentistry, enabled by the widespread smartphone adoption, uses information and communication technologies to deliver remote care, expand access in rural and geographically disadvantaged areas, and support health promotion, monitoring, and triage.^419^, ^420^, ^421^, ^422^ Artificial intelligence (AI), defined as the capability of machines to perform intellectual tasks, which traditionally were assumed exclusive to humans,^423^ enabling risk prediction, early detection from clinical images, and decision support to personalize preventive care. A summary of the 39 included studies is presented in Table 1.

**Table 1:** Summary of digital applications in preventive dentistry (n = 39).

Indications	Digital technologies	Key applications in studies
Primary prevention	Digital imaging (photographs)	• Visualization of oral hygiene^32^^,^^33^
	Teledentistry	• Mobile applications coupled with toothbrushes^28^, ^29^, ^30^, ^31^ • Mobile applications for oral hygiene instruction^15^, ^16^, ^17^, ^18^, ^19^, ^20^, ^21^, ^22^, ^23^, ^24^, ^25^, ^26^, ^27^ • Reminder messages of oral hygiene practice^11^, ^12^, ^13^, ^14^
Secondary prevention	AI	• Automatic diagnosis of oral lesions and dental pain^40^, ^41^, ^42^, ^43^, ^44^, ^45^
	Digital imaging (IOS)	• Monitoring progression of tooth wear^46^, ^47^, ^48^, ^49^
	Teledentistry	• Remote diagnosis of common oral conditions^34^, ^35^, ^36^, ^37^, ^38^, ^39^

AI, artificial intelligence; IOS, intraoral scanner.

##### Primary prevention

Primary prevention aims to prevent disease from becoming established by eliminating its causes. In teledentistry, a foundational application is delivering smartphone-based oral hygiene reminders.^11^, ^12^, ^13^, ^14^ Building on this, mobile applications use multimodal strategies to promote proper oral hygiene and have shown effectiveness across age groups.^15^, ^16^, ^17^, ^18^, ^19^, ^20^, ^21^, ^22^, ^23^, ^24^, ^25^, ^26^, ^27^ When integrated with sensor-equipped toothbrushes, these apps can significantly improve brushing behaviours and oral hygiene.^28^, ^29^, ^30^, ^31^ Longer-term studies are needed to determine whether these digital interventions translate into measurable reductions in disease incidence and prevalence. Digital imaging, including intraoral cameras, further supports primary prevention by enabling personalized visualization of the oral cavity and assessment of oral hygiene.^32^^,^^33^

##### Secondary prevention

Secondary prevention targets early detection of diseases to enable timely intervention and disease control. Digital imaging and telecommunication support remote sharing of intraoral photographs, radiographs, and video recordings for diagnosis and treatment planning,^34^, ^35^, ^36^, ^37^, ^38^, ^39^ benefiting individuals with limited mobility or chronic conditions. AI further advances early detection: AI-powered mobile health tools can analyse oral mucosal lesions and other pathologies with diagnostic performance comparable to experienced clinicians.^40^, ^41^, ^42^, ^43^, ^44^, ^45^ Digital tools also facilitate the monitoring of ongoing conditions – using intraoral scanners (IOS), clinicians can rapidly generate digital models of the dentition to monitor tooth wear progression over time.^46^, ^47^, ^48^, ^49^

##### Tertiary prevention

Tertiary prevention focuses on the management and rehabilitation of patients with established dental conditions to restore function and improve quality of life. Related strategies are discussed in the section Management of disease consequence and patient care.

In conclusion, digital technologies – particularly teledentistry and artificial intelligence – are integral to preventive dentistry, supporting primary prevention through digital education and behavior-change tools and enabling secondary prevention through early disease detection and remote monitoring.

##### Cariology

Digital technologies are increasingly integrated into cariology, with applications in prevention, detection, and risk assessment of dental caries, as summarized in Table 2 (26 articles).

**Table 2:** Summary of digital applications in cariology (n = 26).

Indications	Digital technologies	Key applications in studies
Prevention	AI	• Customized hygiene promotion for caries reduction^52^
	Teledentistry	• Hygiene promotion for caries reduction^50^^,^^51^
Diagnosis	AI	• Automated diagnosis based on radiographs, photographs and IOS^63^, ^64^, ^65^, ^66^, ^67^, ^68^, ^69^, ^70^, ^71^, ^72^
	Teledentistry	• Remote diagnosis based on photographs^53^, ^54^, ^55^, ^56^, ^57^, ^58^, ^59^, ^60^, ^61^, ^62^
Risk prediction	AI	• Caries risk prediction based on demographical and clinical metrics^73^, ^74^, ^75^

AI, artificial intelligence; IOS, intraoral scanner.

##### Caries prevention

Teledentistry interventions delivering reminder messages and educational content can promote oral health behaviours, resulting in short-term increases in tooth brushing frequency; however, these effects have not been sustained over time and have not effectively prevented caries.^50^, ^51^, ^52^

##### Caries diagnosis

Teledentistry enables remote caries assessment via intraoral or phone photographs, with accuracy comparable to clinical examination.^53^, ^54^, ^55^, ^56^, ^57^, ^58^, ^59^, ^60^, ^61^, ^62^ The integration of AI further enhances the automated detection using intraoral photos, radiographs or digital scans.^63^, ^64^, ^65^, ^66^, ^67^, ^68^, ^69^, ^70^, ^71^, ^72^

##### Caries risk assessment

AI-driven caries risk assessment models trained on behavioural determinants can predict early childhood caries risk and support targeted personalized preventative recommendations.^73^, ^74^, ^75^

##### Periodontology

Across the 16 periodontology articles included (Table 3), digital technologies are used to enhance the prevention, detection, monitoring, and management of periodontal disease.

**Table 3:** Summary of digital applications in periodontology (n = 16).

Indications	Digital technologies	Key applications in studies
Prevention	Digital imaging (IOS)	• Gingival inflammation monitoring and hygiene promotion^76^^,^^77^
Detection and diagnosis	AI	• Alveolar bone level detection based on radiographs^80^, ^81^, ^82^, ^83^ • Gingivitis detection based on photographs^78^^,^^79^
	Electronic periodontal probe	• Pocket depth measurements^84^, ^85^, ^86^
Risk prediction	AI	• Periodontal disease prediction based on demographical and clinical metrics^87^, ^88^, ^89^, ^90^, ^91^

AI, artificial intelligence; IOS, intraoral scanner.

##### Prevention of periodontal disease

For prevention, IOS captures high-quality, true-colour images that reflect gingival health, demonstrating 90% agreement with clinical assessments of gingival inflammation.^76^ IOS-derived data can inform personalized hygiene advice and reminder-based interventions which improved bleeding on probing and plaque scores over 6 months.^77^

##### Detection and diagnosis of periodontal disease

AI systems trained on photographic and radiographic data can identify gingivitis, quantify alveolar bone loss, and detect intrabony defects, supporting periodontitis staging.^78^, ^79^, ^80^, ^81^, ^82^, ^83^ Electronic periodontal probes that apply calibrated pressure achieve less than 0.5 mm deviation relative to manual probing, providing a reliable digital alternative.^84^, ^85^, ^86^

##### Risk prediction

Machine-learning models leveraging large-scale electronic records have been developed to predict periodontal disease and tooth-loss phenotypes.^87^, ^88^, ^89^, ^90^, ^91^ These advancements have the potential to reduce workload and enable data-driven dental care.

#### Management of disease consequences and patient care

The section focuses on treatment workflows – from digital planning to precision surgery and computer-aided manufacturing – designed to improve outcomes related to disease consequences. These studies account for approximately 80% of the review and span prosthodontics, oral and maxillofacial surgery, orthodontics, and perioperative management.

##### Prosthodontics

Digital applications in prosthodontics – including CAD-CAM and digital imaging – enhance accuracy and predictability in prosthesis fabrication and dental rehabilitation.

Computer-aided design (CAD) and computer-aided manufacturing (CAM) systems comprise 3 main components: digital patient data as input, a CAD system for electronic modelling and planning, and a CAM system for automated fabrication of dental appliances.^424^ CAD systems integrate multiple digital data modalities to create a 3D virtual patient that replicates the aesthetic and functional characteristics of the real patient.^425^, ^426^, ^427^ Intraoral structures can be digitized indirectly by scanning stone casts or directly using IOS.^139^^,^^428^^,^^429^ Facial scanners, along with virtual facebows and jaw trackers, capture 3D facial morphology and mandibular movements.^430^, ^431^, ^432^, ^433^, ^434^, ^435^ Cone beam computed tomography (CBCT) enables 3-dimensional reconstruction of maxillofacial bone structures.^436^ A summary of these applications is provided in Table 4 (127 articles).

**Table 4:** Summary of digital applications in prosthodontics (n = 127).

Indications	Digital technologies	Key applications in studies
Treatment planning	CAD-CAM and AI	• Digital smile design^94^, ^95^, ^96^, ^97^ • Restorations design^98^, ^99^, ^100^
Tooth preparation	CAD-CAM	• Customized implant abutment fabrication^107^, ^108^, ^109^, ^110^, ^111^, ^112^^,^^218^ • Template-assisted tooth preparation^106^
Impression, occlusion and teeth-to-face relationships	CAD-CAM	• Customized impression trays^134^
	Digital imaging	• Digital impression for implants^115^, ^116^, ^117^, ^118^, ^119^, ^120^, ^121^^,^^127^^,^^128^
		• Digital impression for teeth^113^^,^^114^^,^^122^, ^123^, ^124^, ^125^, ^126^^,^^129^, ^130^, ^131^, ^132^, ^133^
		• Digital occlusal records^135^, ^136^, ^137^
		• Virtual mounting^138^
Prosthesis fabrication	CAD-CAM	• Fixed prostheses^150^, ^151^, ^152^, ^153^, ^154^, ^155^^,^^161^, ^162^, ^163^, ^164^^,^^170^, ^171^, ^172^, ^173^, ^174^, ^175^, ^176^, ^177^, ^178^, ^179^, ^180^, ^181^, ^182^, ^183^, ^184^, ^185^, ^186^, ^187^^,^^217^ • Implant-supported prostheses^139^, ^140^, ^141^, ^142^, ^143^, ^144^, ^145^, ^146^, ^147^, ^148^, ^149^^,^^158^, ^159^, ^160^^,^^165^, ^166^, ^167^^,^^190^, ^191^, ^192^, ^193^, ^194^, ^195^, ^196^, ^197^, ^198^, ^199^, ^200^, ^201^, ^202^, ^203^, ^204^, ^205^, ^206^, ^207^, ^208^, ^209^ • Removable prostheses^156^^,^^157^^,^^168^^,^^169^^,^^188^^,^^189^
Miscellaneous		
Crown lengthening surgery	CAD-CAM and CAS	• Surgical guides^210^^,^^211^
Shade selection	Digital imaging	• Digital shade selection using IOS^103^, ^104^, ^105^ • Digital shade selection using smartphone^101^^,^^102^
Temporomandibular disorder	AI	• Prediction model based on demographic and medical metrics^215^^,^^216^
	CAD-CAM	• Occlusal devices^212^, ^213^, ^214^
	Teledentistry	• Digital therapeutics consisting of education, self-exercise and monitoring^92^^,^^93^

AI, artificial intelligence; CAD-CAM, computer-aided design-computer-aided manufacturing; CAS, computer-assisted surgery; IOS, intraoral scanner.

##### Treatment planning

Advancements in Digital Smile Design (DSD) – integrating intraoral scanning, facial scanning, and CAD – allow clinicians to visualize and plan restorations in 3D, improving predictability and patient communication.^94^, ^95^, ^96^, ^97^ In CAD systems, restoration morphology can be designed using either the correlation method or the library method.^98^^,^^99^ AI-powered prosthetic design has also emerged, using deep learning algorithms to generate precise and biomimetic restoration morphology.^100^

##### Tooth preparation

Tooth preparation benefits from CAD-CAM-generated templates that support precise and controlled tooth reduction.^106^ In implant prosthodontics, digitally fabricated abutments provide customized solutions for diverse clinical scenarios, with high survival rates and stable peri-implant tissues.^107^, ^108^, ^109^, ^110^, ^111^, ^112^^,^^218^

##### Impression, occlusion and teeth-to-face relationships

The shift from conventional to digital impressions reduces distortions associated with impression materials and improves patient comfort. Advances in IOS, including powder-free scanning and faster acquisition, have made them increasingly preferred by clinicians and patients, while maintaining accuracy and restoration quality.^113^, ^114^, ^115^, ^116^, ^117^, ^118^, ^119^, ^120^, ^121^, ^122^, ^123^, ^124^, ^125^, ^126^, ^127^, ^128^

Despite these advantages, digital impressions can be challenging in edentulous patients.^129^ To address this limitation, alternative methods such as stereophotogrammetry and IOS with auxiliary structures have been introduced.^130^, ^131^, ^132^, ^133^ Digital methods streamline occlusal records and the establishment of teeth-to-face relationships, achieving outcomes comparable to conventional techniques with reduced adjustment time.^134^, ^135^, ^136^, ^137^, ^138^

##### Prosthesis fabrication

The fabrication process in prosthodontics has shifted from traditional manual techniques to CAD-CAM-based subtractive and additive manufacturing. These digital approaches enhance restoration accuracy, reduce fabrication time, and improve cost-effectiveness for fixed and removable prostheses, including implant-supported restorations.^139^, ^140^, ^141^, ^142^, ^143^, ^144^, ^145^, ^146^, ^147^, ^148^, ^149^, ^150^, ^151^, ^152^, ^153^, ^154^, ^155^, ^156^, ^157^, ^158^, ^159^, ^160^, ^161^, ^162^, ^163^, ^164^, ^165^, ^166^, ^167^, ^168^, ^169^, ^170^, ^171^, ^172^, ^173^, ^174^, ^175^, ^176^, ^177^, ^178^, ^179^, ^180^, ^181^, ^182^, ^183^, ^184^, ^185^, ^186^, ^187^, ^188^, ^189^, ^190^, ^191^, ^192^, ^193^, ^194^, ^195^, ^196^, ^197^, ^198^, ^199^, ^200^, ^201^, ^202^, ^203^, ^204^, ^205^, ^206^, ^207^, ^208^, ^209^^,^^217^

##### Miscellaneous

3D-printed surgical guides for crown lengthening enable shorter surgery times, improved aesthetic outcomes, and better soft tissue stability.^210^^,^^211^ Tooth shade selection is a critical yet challenging step in prosthodontics. Traditionally, it has been subjective and prone to inconsistencies. Digital methods, including IOS and AI-assisted mobile applications, aim to reduce subjectivity and improve consistency, supporting more predictable aesthetic outcomes.^101^, ^102^, ^103^, ^104^, ^105^ Both additively and subtractively manufactured occlusal devices provide comparable therapeutic effects while reducing wear of antagonist teeth as well as device wear.^212^, ^213^, ^214^ AI-driven prediction models have been developed to identify temporomandibular disorders,^215^^,^^216^ and digital therapeutics delivered via mobile applications have shown improved outcomes for patients with temporomandibular disorders.^92^^,^^93^

From shade selection to prosthetic design and fabrication, digital technologies have enhanced restoration precision and longevity while minimizing procedural errors. Continued adoption will further refine prosthodontic workflows, making them more precise, cost-effective, and accessible.

##### Oral & maxillofacial surgery

The integration of digital technologies – particularly computer-assisted surgery (CAS) and CAD-CAM systems – has enhanced the accuracy and predictability of diverse procedures, as summarized in the 112 articles included in Table 5. CAS encompasses 2 approaches: static computer-assisted surgery (SCAS), which uses a fixed surgical template,^437^ and dynamic computer-assisted surgery (DCAS) which employs optical motion-tracking for real-time guidance.^438^, ^439^, ^440^, ^441^

**Table 5:** Summary of digital applications in oral & maxillofacial surgery (n = 112).

Indications	Digital technologies	Key applications in studies
Surgical planning	AI	• Surgical risks prediction based on radiographs and medical metrics^221^^,^^222^
	Digital imaging and CAD-CAM	• Digital surgical planning in virtual patient^223^, ^224^, ^225^, ^226^, ^227^
Bone augmentation	CAD-CAM	• 3D-printed bone grafts^236^ • 3D-printed meshes^237^^,^^238^ • Simulation model^239^, ^240^, ^241^
	CAS	• Surgical guides or navigation^242^, ^243^, ^244^, ^245^, ^246^, ^247^, ^248^
Dental implantology	AI	• Automated treatment planning^249^ • Implant classification based on radiographs^250^, ^251^, ^252^
	CAD-CAM	• Customized endosseous implants^253^^,^^254^ • Customized healing abutments^255^, ^256^, ^257^, ^258^ • Customized subperiosteal implants^259^^,^^260^
	CAS	• Dynamic navigation-assisted implant placement^261^, ^262^, ^263^, ^264^, ^265^, ^266^, ^267^, ^268^, ^269^, ^270^ • Template-guided implant placement^264^^,^^265^^,^^269^, ^270^, ^271^, ^272^, ^273^, ^274^, ^275^, ^276^, ^277^, ^278^, ^279^, ^280^, ^281^, ^282^, ^283^, ^284^, ^285^, ^286^, ^287^, ^288^, ^289^, ^290^, ^291^, ^292^, ^293^, ^294^, ^295^, ^296^, ^297^, ^298^, ^299^, ^300^, ^301^, ^302^
	Robot	• Robot-assisted implant placement^303^, ^304^, ^305^, ^306^, ^307^
Dentoalveolar surgery	CAS	• Impacted teeth extractions and eruption^228^, ^229^, ^230^
Maxillofacial surgery	CAS	• Bone fracture treatment^314^, ^315^, ^316^ • Fibula reconstruction^317^, ^318^, ^319^, ^320^ • Naso-alveolar molding^321^ • Orthognathic surgery^322^, ^323^, ^324^, ^325^, ^326^, ^327^, ^328^ • Resection surgery^329^^,^^330^
Miscellaneous		
Autotransplantation	Digital imaging (CBCT) and CAD-CAM	• Tooth replicas^231^, ^232^, ^233^, ^234^, ^235^
Head and neck cancer	AI	• Survival and recurrence prediction based on demographic and medical metrics^311^^,^^312^
	CAD-CAM	• Customized stents for radiotherapy^313^
Postsurgery	Teledentistry	• Dentoalveolar surgery aftercare^308^, ^309^, ^310^
Trigeminal neuralgia	CAD-CAM	• Guided templates^219^^,^^220^

AI, artificial intelligence; CAD-CAM, computer-aided design-computer-aided manufacturing; CAS, computer-assisted surgery; CBCT, cone-beam computed tomography.

##### Surgical planning

High-resolution 3D imaging underpins digital planning, reducing linear and angular errors.^223^, ^224^, ^225^, ^226^, ^227^ AI-driven models also assist in predicting surgical risks and postoperative pain accurately in third molar removal.^221^^,^^222^

##### Bone augmentation

In guided bone regeneration (GBR), CAD-CAM supports planning and the fabrication of surgical guides for localized defects.^237^, ^238^, ^239^, ^240^, ^241^ Advances in 3D printing enable customized bone grafts tailored to specific defect morphology.^236^ Surgeons can also 3D print guides and use dynamic navigation for intraoral block bone grafting, ridge splitting, cyst aspiration, and sinus floor augmentation.^242^, ^243^, ^244^, ^245^, ^246^, ^247^, ^248^

##### Dental implantology

Both SCAS and DCAS reduce surgical deviations compared with conventional approaches.^261^, ^262^, ^263^, ^264^, ^265^, ^266^, ^267^, ^268^, ^269^, ^270^, ^271^, ^272^, ^273^, ^274^, ^275^, ^276^, ^277^, ^278^, ^279^, ^280^, ^281^, ^282^, ^283^, ^284^, ^285^, ^286^, ^287^, ^288^, ^289^, ^290^, ^291^, ^292^, ^293^, ^294^, ^295^, ^296^, ^297^, ^298^, ^299^, ^300^, ^301^, ^302^ Computer-assisted implant placement can lessen the need for bone augmentation procedures and reduce treatment complexity.^301^ Comparative studies indicate similar postoperative pain and swelling among static, dynamic, and freehand techniques.^269^^,^^270^ Robot-assisted implant placement as an emerging option that can further improve precision,^303^, ^304^, ^305^, ^306^, ^307^ though limited tactile feedback may affect the accuracy of self-tapping implant insertion.^304^ Customized healing abutments improve soft- and hard-tissue preservation postoperatively.^255^, ^256^, ^257^, ^258^ CAD-CAM systems enable fabrication of custom endosseous and subperiosteal implants for atrophic jaws.^253^^,^^254^^,^^259^^,^^260^ Deep learning models have also been developed for automated treatment planning and dental implant classification.^249^, ^250^, ^251^, ^252^

##### Dentoalveolar surgery

CAS aids precise localization and eruption trajectory control for target teeth, improving accuracy and efficiency. Digital guides and 3D imaging reduce operative time and postoperative pain, and minimize bone removal for extractions and for orthodontic eruption of impacted teeth.^228^, ^229^, ^230^

##### Maxillofacial surgery

Digital precision extends to maxillofacial surgical procedures.^329^^,^^330^ 3D-printed occlusal splints and surgical templates derived from virtual simulation guide osteotomy lines and skeletal movements.^322^, ^323^, ^324^, ^325^, ^326^, ^327^, ^328^ In cleft lip and palate, 3D-printed maxillary models facilitate effective naso-alveolar molding,^321^ while for fractures and mandibular defect reconstruction, CT-based virtual models and CAD-CAM-customized guides or plates enable precise alignment and stable bone healing.^314^, ^315^, ^316^, ^317^, ^318^, ^319^, ^320^

##### Miscellaneous

Additional applications include autotransplantation with CBCT replicas for precise socket preparation,^231^, ^232^, ^233^, ^234^, ^235^ 3D-printed oral stents for radiotherapy protection,^313^ and personalized templates for minimally invasive trigeminal neuralgia treatment.^219^^,^^220^ Telemedicine has demonstrated effectiveness for postoperative follow-up in dentoalveolar surgery.^308^, ^309^, ^310^ Machine learning models may assist in predicting survival and recurrence risks in oral cancer.^311^^,^^312^

Overall, digital technologies are improving surgical accuracy, efficiency, and consistency in oral and maxillofacial surgery, with corresponding gains in clinical outcomes.

##### Orthodontics

Teledentistry, AI, and CAD-CAM systems have made substantial contributions to orthodontic care, offering clinicians innovative tools to improve treatment planning and patient communication, as outlined in Table 6 (26 articles).

**Table 6:** Summary of digital applications in orthodontics (n = 26).

Indications	Digital technologies	Key applications in studies
Treatment planning	AI	• Diagnosis and planning^333^, ^334^, ^335^, ^336^
Treatment outcome simulation	Digital imaging, CAD-CAM, and AI	• Predictive digital model^337^, ^338^, ^339^, ^340^
Appliances fabrication	CAD-CAM	• Bracket system^341^ • Guided bonding devices^342^^,^^343^ • Orthodontic aligners^344^ • Retainers^345^ • Space maintainers^346^
Patient management	Teledentistry	• Compliance monitoring^350^ • Oral health promotion in orthodontics^351^, ^352^, ^353^, ^354^, ^355^, ^356^ • Referral assessment^331^^,^^332^
Miscellaneous		
Corticotomy	CAD-CAM	• Surgical guides^347^^,^^348^
Palatal expansion	CAS	• Miniscrew insertion^349^

AI, artificial intelligence; CAD-CAM, computer-aided design-computer-aided manufacturing; CAS, computer-assisted surgery.

##### Treatment planning

AI now supports semi-automatic and fully automated cephalometric analyses.^334^^,^^335^ It has also been leveraged for malocclusion classification using fully convolutional neural network applied to intraoral photographs.^336^ AI-enhanced diagnostic tools have been associated with shorter treatment times, higher planning accuracy, and higher patient satisfaction compared with traditional methods.^333^

##### Treatment outcomes simulation

3D digital models enable patients to visualize predicted outcomes, helping set realistic expectations and improving treatment understanding and satisfaction.^337^, ^338^, ^339^ SmileView allows users to upload a selfie and receive an instant, AI-powered simulation of their potential smile transformation.^340^

##### Appliances fabrication

CAD-CAM and 3D printing enable precise, patient-specific appliances – including space maintainers, bracket system, removable aligners, and guided bonding devices – improving fit, efficiency, and clinical outcomes.^341^, ^342^, ^343^, ^344^^,^^346^ However, CAD-CAM retainers experienced a 50% failure rate within 6 months, possibly due to manufacturing delays leading to complications and suboptimal outcomes.^345^

##### Patient management

Teledentistry streamlines referrals by enabling clinicians to forward radiographs and clinical data to specialists, reducing unnecessary visits.^331^^,^^332^ It also supports patient education and engagement through reminders and educational clips, while applications like Dental Monitoring allow patients to submit photos for AI-assisted assessment of tooth movement and hygiene, reducing in-person appointments.^351^, ^352^, ^353^, ^354^, ^355^, ^356^ A Bluetooth-connected retainer can synchronize with smartphones to monitor patient compliance.^350^

##### Miscellaneous

Digital workflows also support surgical adjuncts, such as 3D-printed guides for piezoelectric corticotomies,^347^^,^^348^ and dynamic navigation for miniscrew insertion for palatal expansion.^349^

Overall, the integration of teledentistry, AI models, and CAD-CAM systems has advanced orthodontics by enabling more accurate diagnoses, better treatment planning, and improved patient communication.

##### Perioperative management

The section focuses on perioperative patient management in dental care – including preoperative, intraoperative and postoperative care – drawing on 61 articles (15%) summarized in Table 7.

**Table 7:** Summary of digital applications in perioperative management (n = 61).

Indications	Digital technologies	Key applications in studies
Preoperative management	AI (large language model)	• Automatic consultation and education on dental conditions, eg, periodontology, dental implants, orthodontics, etc.^361^, ^362^, ^363^, ^364^, ^365^, ^366^
Intraoperative management	Computer-controlled delivery systems	• Local anesthesia^367^, ^368^, ^369^, ^370^, ^371^
	Mobile games	• Paediatric behaviour guidance^372^, ^373^, ^374^, ^375^, ^376^, ^377^, ^378^
	VR and electronic devices	• Distraction technique to alleviate pain and distress^379^, ^380^, ^381^, ^382^, ^383^, ^384^, ^385^, ^386^, ^387^, ^388^, ^389^, ^390^, ^391^, ^392^, ^393^, ^394^, ^395^, ^396^, ^397^, ^398^, ^399^, ^400^, ^401^, ^402^, ^403^, ^404^, ^405^, ^406^, ^407^, ^408^, ^409^, ^410^, ^411^, ^412^, ^413^, ^414^, ^415^, ^416^
Postoperative management	AI	• Postoperation responses prediction based on demographic and medical metrics^417^
	Teledentistry	• Customized monitoring and guidance of pain and complications^357^, ^358^, ^359^, ^360^

AI, artificial intelligence; VR, virtual reality.

##### Preoperative management

Large language model-based chatbots can provide patient-centre support for preliminary consultation, screening, and education on issues related to periodontal care, dental implants, orthodontics, radiology, and more.^361^, ^362^, ^363^, ^364^, ^365^, ^366^

##### Intraoperative management

During dental procedures, virtual reality (VR) immerses patients in simulated environments and has been shown to be an effective distraction technique that helps manage dental phobia and alleviate pain and distress.^383^, ^384^, ^385^, ^386^, ^387^, ^388^, ^389^, ^390^, ^391^, ^392^, ^393^, ^394^, ^395^, ^396^, ^397^, ^398^, ^399^, ^400^, ^401^, ^402^, ^403^, ^404^, ^405^, ^406^, ^407^, ^408^, ^409^, ^410^, ^411^, ^412^, ^413^, ^414^, ^415^, ^416^ However, some individuals exhibited higher heart rates with VR than with cartoon videos, suggesting that VR may induce stress in certain contexts, particularly when patients feel isolated or lacks control.^382^ Other electronic devices have also been shown to reduce anxiety.^379^, ^380^, ^381^ During local anaesthesia, computer-controlled anaesthetic delivery systems regulate injection rate and pressure, resulting in less pain and anxiety than manual injections.^367^, ^368^, ^369^, ^370^, ^371^ In paediatric behaviour guidance, mobile dental games that simulate treatment in a playful manner are more effective than the traditional tell-show-do method in reducing anxiety and improving cooperation.^372^, ^373^, ^374^, ^375^, ^376^, ^377^, ^378^

##### Postoperative management

Teledentistry supports remote monitoring and management of postoperative dental pain and complications, enhancing communication and continuity of care.^359^^,^^360^ After periodontal treatment, mobile applications and intelligent power-driven toothbrushes can track periodontal parameters, deliver hygiene instructions, and improve plaque control.^357^^,^^358^ Machine learning has been used to predict post-treatment responses, enabling more personalized treatment plans.^417^

Overall, digital technologies are enhancing patient experience and satisfaction across all stages of care, from preoperative to postoperative.

This scoping review comprehensively synthesized the clinical applications of digital dentistry across multiple dental disciplines within the scope of the FDI Policy Statement. It shares the same goal of encouraging dental professionals, educators, researchers, and policymakers to embrace advancements while addressing the associated challenges. Moreover, it provides up-to-date and detailed insights to support readers in understanding and implementing the FDI Policy Statement in practice.

However, several limitations warrant considerations. First, restricting inclusion to English-language publications may have introduced selection bias. Second, to maintain a focused scope on clinical outcomes, we excluded educational and other nonclinical studies; consequently, some preclinical digital technologies with substantial promises were not examined. Third, the breadth of disciplines and technologies represented resulted in substantial heterogeneity in study designs, populations, methodologies, and study quality, which precluded direct comparisons and may have biased assessments of clinical effectiveness. These factors temper the generalizability of our findings.

## Conclusion

This review shows that digital dentistry now spans a broad suite of technologies that improve efficiency, accuracy, and care quality across multiple dental disciplines. As novel tools and indications emerge, its scope will continue to grow, becoming more comprehensive and integral to routine practice. Realizing this potential will require addressing challenges in evidence generation and validation, workflow integration and interoperability, data security and ethics, training and change management, and cost and equitable access. In line with the FDI Policy Statement, the following recommendations are proposed for dental professionals, educators, and policymakers:

• Align digital dentistry with primary healthcare and global oral health strategies.
• Critically evaluate the evidence supporting digital dentistry applications.
• Promote user-friendly technologies that are accessible to both providers and patients alike.
• Enhance education and training to enable effective use of digital technologies while retaining professional judgement and responsible patient management.
• Integrate comprehensive digital dentistry curricula across all levels of dental education.
• Uphold legal and regulatory frameworks that protect privacy and ensure secure data collection, storage, and appropriate access.
• Support the development and adoption of relevant standards to ensure the quality, effectiveness, safety, interoperability, and applicability of digital dentistry.

## Author contributions

W.L. contributed to conception, design, and critically revised manuscript. Z.L. contributed to conception, design, data acquisition, interpretation, drafted and critically revised manuscript. K.M. contributed to data acquisition and interpretation. J.P., A.Z., A.C., F.M., J.P. and F.S. contributed to conception, and critically revised manuscript. All authors gave final approval and agreed to be accountable for all aspects of the work.

## Conflict of interest

The authors declare that they have no known competing financial interests or personal relationships that could have appeared to influence the work reported in this paper.
